# Cervical Cancer Cells Use the CD95 and IL-2 Pathways to Promote Their Proliferation and Survival

**DOI:** 10.3390/biom14121543

**Published:** 2024-12-01

**Authors:** Adriana Gutiérrez-Hoya, Ivan Ortiz-Garrido, Itzel Salazar-Valencia, Christopher Romero-Hernández, Arturo Valle-Mendiola, Benny Weiss-Steider, Isabel Soto-Cruz

**Affiliations:** 1 Molecular Oncology Laboratory, Cell Differentiation and Cancer Research Unit, UMIEZ Campus II FES Zaragoza, National Autonomous University of Mexico, Mexico City 09230, Mexico; agutierrezho@conacyt.mx (A.G.-H.); iv_orga@hotmail.com (I.O.-G.); itzviolista@gmail.com (I.S.-V.); christopher.romeroh@gmail.com (C.R.-H.); arturo.valle@zaragoza.unam.mx (A.V.-M.); bennyweiss@hotmail.com (B.W.-S.); 2 Researcher CONAHCYT, Consejo Nacional de Humanidades, Ciencias y Tecnologías, Col. Crédito Constructor, Benito Juárez, Mexico City 03940, Mexico; 3 Population Genomics Unit Applied to Health, Faculty of Chemistry, National Autonomous University of Mexico, Mexico City 14610, Mexico; 4 Postgraduate Program in Biological Sciences, Postgraduate Unit, Building D, 1st Floor, University City, National Autonomous University of Mexico, Coyoacán, Mexico City 04510, Mexico

**Keywords:** cervical cancer, interleukin-2, CD95, cell proliferation, agonist antibodies, LC3B foci

## Abstract

Cervical cancer is a global health problem; therapies focused on eliminating tumour cells and strengthening different immunotherapies are in development. However, it has been observed that cervical tumour cells can evade cell death mechanisms and generate immune system molecules to promote their proliferation and metastasis. In this context, we analysed the role of the IL-2 and CD95 pathways, essential molecules in activating the immune system and eliminating tumour cells. However, it is important to analyse their role in cervical tumour cells because these cells could be using these pathways to proliferate. In this study, we found that SiHa and HeLa cells respond to treatment, with 10 IU/mL of IL-2 inducing their proliferation and 100 IU/mL of IL-2 decreasing their proliferation. We also observed that they express a high percentage of the CD95 receptor and its ligand (CD95L) and that treatment with CD95 agonist antibodies at low doses increases cell proliferation. Furthermore, simultaneous treatment with high doses of IL-2 plus CD95 agonist antibody positively regulates LC3B accumulation. We did not observe apoptosis under any of the treatments carried out. In conclusion, cervical tumour cells can use the IL-2 and CD95 pathways to induce their proliferation and potentially activate cytoprotective mechanisms for survival.

## 1. Introduction

Cervical cancer is associated with persistent infection with high-risk Human Papillomavirus (HPV), mainly HPV16+ and HPV18+ [1]. The activation of cell death plays a vital role in promoting the elimination of damaged, infected, and transformed cells [2]. It has been reported that cervical tumour cells have a high expression of the death receptor CD95 [3,4]. However, despite this high expression, HPV-positive cells are highly resistant to the induction of cell death by CD95 [3,5]. In other types of cancer, the CD95/CD95L interaction has been observed to activate the non-canonical pathway that favours resistance to cell elimination and greater motility, invasiveness, proliferation, and survival [6,7]. CD95L has been detected in cervical tumours, which allows them to induce death by the apoptosis of cytotoxic T lymphocytes [8,9]. CD95 is not the only molecule associated with a pleiotropic response; our work group has shown that IL-2 can also have a differential effect on cervical cancer cells. IL-2 is a pleiotropic cytokine related to the activation and regulation of the immune system. It is known as the T cell growth factor and is necessary for activating the immune system. It has been used in the treatment of different types of cancer, such as melanoma and renal cancer. However, due to its toxicity and role in activating regulatory T cells, joint therapies that promote a better immune system activation to eliminate tumour cells are currently sought [10,11]. On the other hand, our work group showed that IL-2 can also be used in an autocrine manner by cervical cancer cells to promote their proliferation [12]. We have previously reported that the treatment of cervical tumour cells with low doses of IL-2 (10 IU/mL) induces increased proliferation. On the contrary, high doses (100 IU/mL) promote a decrease in proliferation due to an apparent arrest in the G1 phase of the cell cycle, possibly promoting a protective effect against death [13]. For this reason, this work aims to analyse the effect of treating cervical tumour cells with IL-2 and their response to death-inducing stimuli such as CD95. The CD95 and IL-2 pathways have also been implicated in activating the apoptotic and autophagy pathways [14,15,16,17]. All of this background led us to analyse the CD95 and IL-2 pathways in cervical cancer cells and determine whether they could have similar behaviour or whether the treatment of both pathways could enhance the activation of apoptosis or the autophagy pathway.

## 2. Materials and Methods

### 2.1. Biological Materials

The HeLa (HPV18; ATCC CCL-2) and SiHa (HPV16; ATCC HTB-35) cell lines were obtained from the American Type Culture Collection (ATCC, Manassas VA, USA). Cervical cancer cells were cultured in RPMI-1640 medium (Microlab, Mexico City, Mexico) adding 10% foetal bovine serum (FBS, Invitrogen, Thermo Fisher Scientific Inc, Waltham, MA, USA). Cell lines were cultured at 37 °C, 5% CO_2_, and saturated humidity in a Sanyo incubator.

### 2.2. Cervical Cancer Cell Treatments

HeLa and SiHa cells were treated with 10 or 100 IU/mL of recombinant human IL-2 (R&D Systems, Minneapolis MN, USA) for 48 h as determined in previous works [12,13], and then restimulated with 10 or 100 IU/mL of IL-2 plus different concentrations of anti-CD95 10, 20, and 25 ng/mL CH11 (Beckman Coulter, Brea CA, USA) or 100, 200, and 400 ng/mL of the DX2 clone (Biolegend, San Diego, CA, USA) for 48 h for combined treatments.

### 2.3. Cell Proliferation Assay

Briefly, 1.5 × 10^3^ HeLa and SiHa cervical cancer cells were cultured on 96-well plates and incubated for 24 h at 37 °C to allow adhesion. Subsequently, the cells were treated with different IL-2 and agonist antibodies anti-CD95 concentrations for 48 and 96 h at 37 °C. For basal proliferation control, HeLa and SiHa tumour cells were cultured for 48 and 96 h without treatment. Once the incubation times had ended, the cells were fixed with 1.1% glutaraldehyde for 20 min (Sigma-Aldrich, Inc. St. Louis, MO, USA). Then, the cells were washed and dried, and the tumour cells were subsequently stained with 0.1% crystal violet (Sigma, USA). Finally, 10% acetic acid was added, and the absorbance was read at 595 nm on a microplate reader (BioRad Laboratories, Hercules, CA, USA).

### 2.4. Flow Cytometry Analyses of Cell Surface and Intracellular CD95 and CD95L of Tumour Cells

To determine CD95 and CD95L on the cell surface, 1 × 10^6^ cervical cancer cells were seeded onto Petri dishes. The cells were stained with specific antibodies, anti-CD95-FITC (152605 Biolegend, San Diego, CA, USA) and anti-CD95L-APC (306421 Biolegend, San Diego, CA, USA), for 35 min at 4 °C in staining buffer (PBS, 0.5% BSA, and 0.01% sodium azide). The cells were washed and fixed with 1% paraformaldehyde for 20 min. To determine the intracellular CD95 and CD95L, the cells were permeabilised using Cytofix/Cytoperm buffer (Becton Dickinson, Franklin Lakes, NJ, USA) for 20 min. The cells were washed and 100 μL of permwash with anti-human CD95-FITC or anti-CD95L-APC antibodies was added. The tumour cells were incubated for 45 min at 4 °C, protected from the light. After the incubation time had ended, the cells were washed and resuspended in staining buffer. Finally, 10,000 events were acquired on an Attune Flow Cytometer (Thermo Fisher Scientific, Inc. Waltham, MA, USA). The data were analysed using Kaluza 2.3 and FlowJo software v10.

### 2.5. Annexin V-FITC and 7AAD Assay

Apoptosis was evaluated by annexin-V and 7AAD staining using the Annexin V-FITC Apoptosis Detection Kit (BD Pharmingen, Franklin Lakes NJ, USA) and 7AAD (BD Pharmingen, Franklin Lakes, NJ, USA). Then, 2.5 × 10^5^ cells were cultured for 24 h; subsequently, the cells were treated with different concentrations of IL-2 and agonist antibodies (anti-CD95) for 48 and 96 h at 37 °C. The cell samples were stained with Annexin V-FITC and 7AAD, following the manufacturer’s protocol. For the negative control, untreated cells were used and for the positive control, cells were treated with 2 μg/mL of cisplatin for 48 h. The percentage of apoptotic cells was determined by flow cytometry using an Attune Flow Cytometer (Thermo Fisher Scientific, Inc. Waltham, MA, USA). The data were analysed using Kaluza and FlowJo software [18,19].

### 2.6. Determination of Extracellular CD95 and Intracellular CD95L by Confocal Microscopy

Approximately 1 × 10^4^ cervical cancer cells were seeded onto glass-bottomed Petri dishes (NEST Biotechnology, Thermo Fisher Scientific, Waltham, MA, USA) and incubated for 24 h. The medium was removed, two washes were carried out with PBS, and then 750 µL PBS was added for staining CD95; 3 µL of anti CD95-FITC was added and incubated at 4 °C in the dark for 2 h. For CD95L, 750 µL of Cytofix/Cytoperm was added, and the samples were incubated for one hour at 4 °C in the dark. Two washes were carried out with Perm wash 1× (750 µL). Subsequently, anti-CD95L-APC (1.5 µL) was added in 750 µL of PBS, and the samples were incubated overnight at 4 °C, protected from light. After the incubation, two washes were performed with 750 μL of 1× perm wash in order to add DAPI (Thermo Fisher Scientific, Inc. Waltham, MA, USA). The samples were incubated for 1.30 h at 4 °C, protected from light. Finally, a wash was carried out using PBS. A mounting medium was added and viewed under a spectral confocal microscope (TCS SP8 Leica Microsystems, Wetzlar, Germany) to acquire the images at a 40× magnification.

### 2.7. LC3B Analysis by Confocal Microscopy

Here, 1 × 10^3^ cells were placed in glass-bottomed dishes (NEST Biotechnology, Thermo Fisher Scientific, Waltham, MA, USA; 15 mm) and incubated for 24 h. The cells were stimulated with 10 IU/mL of IL-2 or 100 IU/mL of IL-2 (R&D Systems, Minneapolis MN, USA) for 48 h prior to further stimulation with 10 or 100 IU/mL of IL-2 plus 20 ng/mL of anti-CD95 (clone CH11, 05-201 Sigma-Aldrich Inc. St. Louis, MO, USA) or 200 ng/mL of clone DX2 (305655, Biolegend, San Diego, CA, USA) in simultaneous treatments. Cisplatin was added as a positive control for another 48 h. The medium was removed, two washes were carried out with PBS, 750 µL of Cytofix/Cytoperm was added, and the samples were incubated while protected from light for one hour at 4 °C. Subsequently, two washes were conducted with Perm wash 1× (750 µL). Then, anti-LC3B (3.75 µL; sc-376404, Santa Cruz Biotechnology Inc., Dallas, TX, USA) was added to 750 µL of PBS and the samples were incubated and protected from light overnight at 4 °C. Subsequently, 1.5 µL of anti-mouse FITC (315-095-003, Jackson Immuno Research Lab., West Grove, PA, USA) was added and incubated. The samples were protected from light for 2 h at 4 °C. Afterwards, two washes were carried out with 750 µL of 1× Perm wash to add DAPI later and incubated for 1.30 h at 4 °C in darkness. Two washes were conducted with PBS; the sample was dried entirely, and a mounting medium was added. Finally, the cells were analysed under a Leica confocal microscope to acquire images at 40× magnification.

### 2.8. Statistical Analysis

Statistical differences between groups were evaluated using one-way analysis of variance (ANOVA). The results are expressed as the mean ± SD. GraphPad Prism 6.0 (GraphPad Software, Inc., San Diego, CA, USA) was used for the analysis. The Bonferroni test was used to correct for multiple comparisons, and *p* values < 0.05 were considered statistically significant.

## 3. Results

### 3.1. CD95/CD95L Are Present in Cervical Tumour Cells

**Figure 1:**
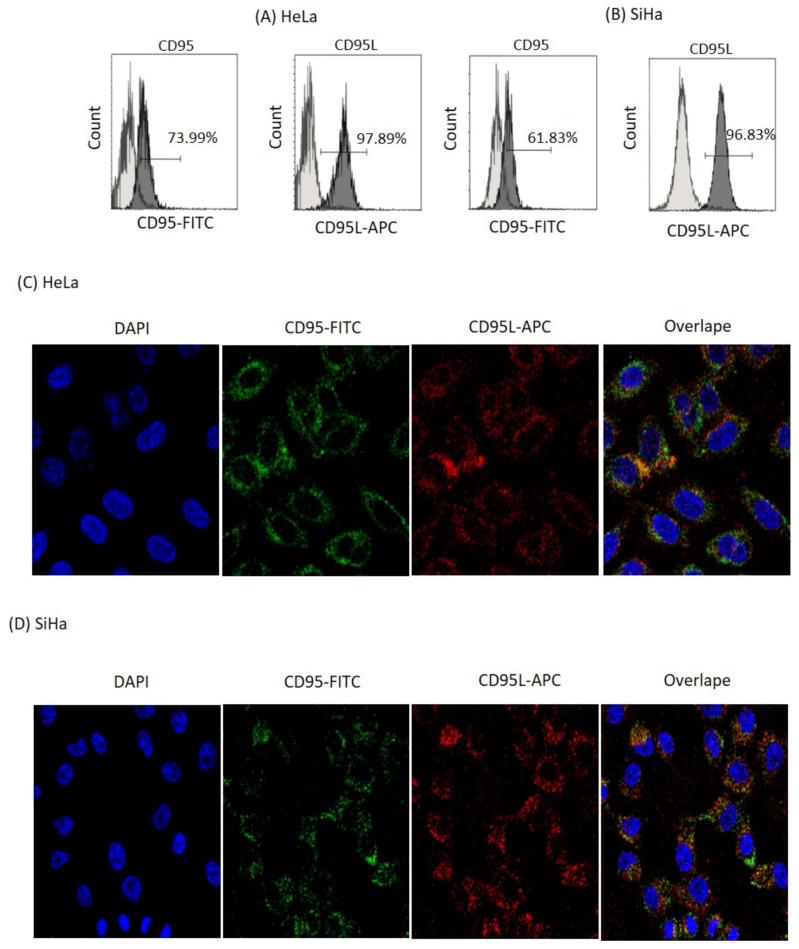
Determination of CD95 and CD95L by flow cytometry and confocal microscopy. (**A**,**B**) Flow cytometry staining of extracellular CD95 and intracellular CD95L in HPV-positive cervical cancer cells. HeLa and SiHa cells express CD95 (61–73%) and CD95L (96–97%); CD95 is coupled to FITC and CD95L to APC. (**C**,**D**) Confocal microscopy staining of cervical tumour cells. HeLa and SiHa express CD95 and CD95L cells. Nuclei are stained with DAPI, FITC-coupled CD95, and APC-coupled CD95L.

HeLa (HPV18) and SiHa (HPV16) cervical tumour cells express the CD95 receptor extracellularly and CD95L intracellularly. The expression of CD95 and its ligand CD95 was analysed in HeLa (HPV18+) and SiHa (HPV16+) cells using flow cytometry (Figure 1A,B) and confocal microscopy (Figure 1C,D). The results show that both cell lines express the marker CD95 (61–73%) and CD95L (96–97%). The simultaneous expression of the receptor and its ligand suggest an interaction, which could activate the non-canonical CD95 pathway and favour mechanisms associated with proliferation, metastasis, and survival [20].

### 3.2. CD95 Induces Cervical Cancer Cell Proliferation

**Figure 2:**
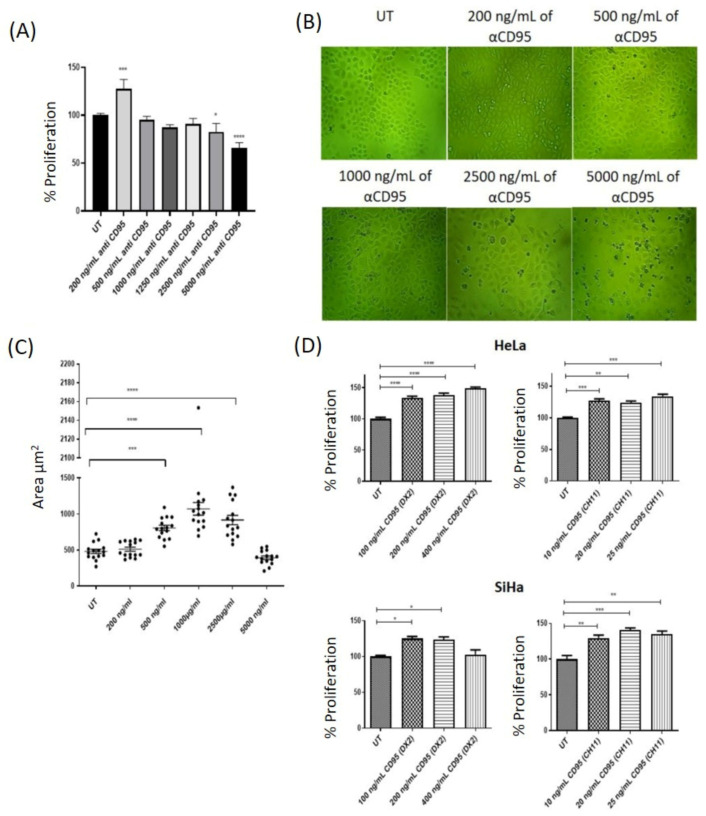
CD95 stimulation in HPV+ cells. (**A**) Cells were treated with 200, 500, 1000, 1250, 2500, and 5000 ng/mL of anti CD95 DX2. The lowest concentration induces cell proliferation, while high concentrations (5000 ng/mL) are needed to decrease cell count. (**B**) Images of cervical tumour cells treated with different anti-CD95 DX2 agonist antibody concentrations. (**C**) Analysis of the cellular area in the tumour cells after treatment with the anti-CD95 antibody. (**D**) Induction of proliferation in HeLa and SiHa cells treated with low concentrations of two different clones of anti-CD95 (DX2 and CH11). The results are expressed as the average. n = 5, * *p* < 0.01, ** *p* < 0.001, *** *p* < 0.0002, and **** *p* < 0.0001.

Cervical tumour cells are resistant to death induced via CD95. However, low doses of the agonist anti-CD95 antibody induce proliferation. Cervical tumour cells were treated with different doses of agonist antibodies to CD95 to determine whether this interaction affected cell proliferation. For this purpose, the anti-CD95 clones DX2 and CH11, which have been observed to be capable of activating the pathway, were used. The results in Figure 2 show that stimulation via CD95 with low concentrations of agonist antibodies (DX2 and CH11) induces cell proliferation. On the contrary, very high doses are required to induce cell death. This effect is clear in HPV18+ cells (HeLa) and HPV16+ (SiHa), which indicates that these cells can use this pathway to induce cell proliferation.

Stimulation of the CD95 pathway using anti-CD95 agonists (CH11, DX2) at low concentrations induces proliferation. It is interesting to note that intermediate concentrations such as 5000 ng/mL of anti-CD95 do not induce an increase in cell proliferation, but do induce an increase in cell size (Figure 2C), which correlates with an increase in fluorescent foci for LC3B (Figures 5 and 6), which indicates that the same stimulus (CD95) could induce proliferation, autophagy, and apoptosis in cervical tumour cells, depending on the dose.

### 3.3. Pleiotropic Effect of CD95 and IL-2 on Cervical Cancer Cells

**Figure 3:**
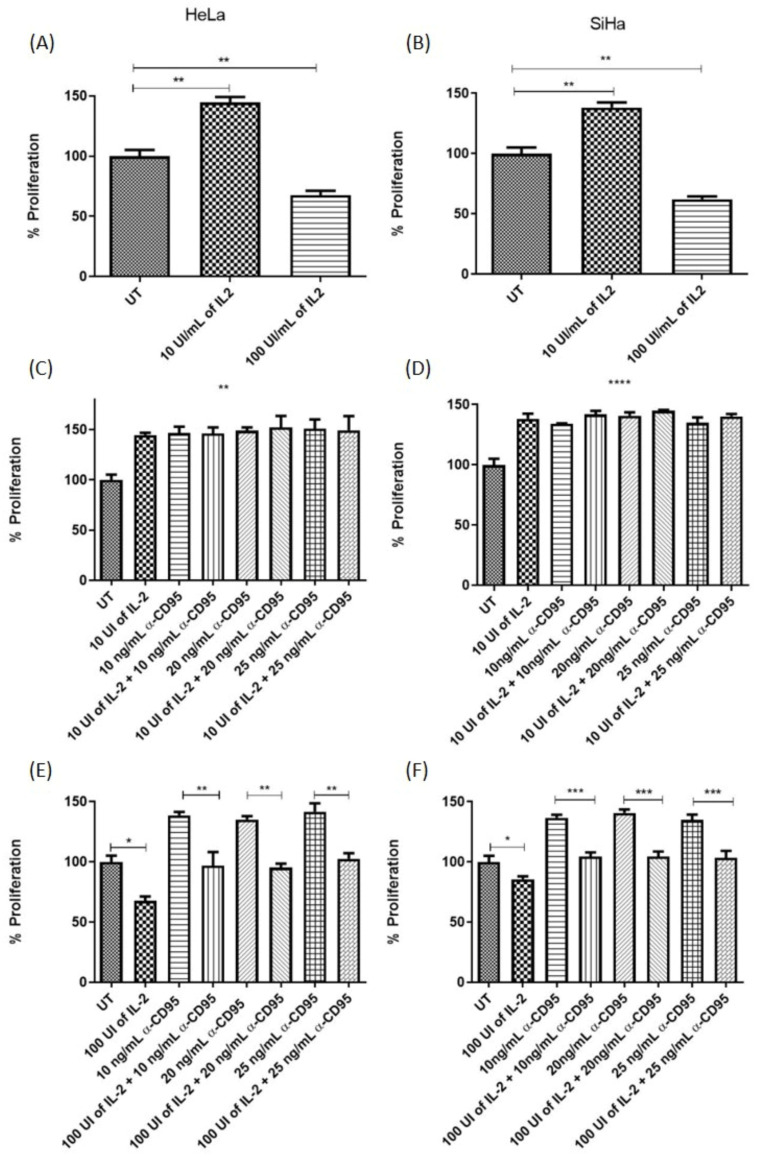
Proliferation analysis of cervical tumour cells treated with IL-2 and IL-2 plus anti-CD95. (**A**,**B**) Treatment of HeLa and SiHa cells with 10 IU/mL of IL-2 induces proliferation; however, treatment of both cell lines with 100 IU/mL of IL-2 inhibits proliferation, and both events are observed up to 30%. (**C**,**D**) HeLa and SiHa cells treated with 10 IU/mL of IL-2 plus 10, 20, or 25 ng of anti-CD95 increase their proliferation compared to the control. (**E**,**F**) Treatment of HeLa and SiHa cells with 100 IU/mL of IL-2 plus 10, 20, or 25 ng of anti-CD95 recovers their proliferation to values similar to those observed in the control. n= 5, * *p* < 0.01, ** *p* < 0.001, *** *p* < 0.0002, and **** *p* < 0.0001.

As shown in Figure 2A, very high doses of the anti-CD95 agonist are needed to reach the IC50 in cervical cancer cells, and with low doses, an induction of proliferation is observed. Another relevant molecule in the control of the immune response is IL-2, which we have previously reported to have a differential effect on cervical cancer cell lines (10). As shown in Figure 3A,B, low doses of IL-2 (10 IU/mL) induce proliferation in HeLa and SiHa cells, while high doses (100 IU/mL) decrease proliferation in these cells. We further evaluated whether pre-treatment with high and low doses of IL-2 affects the response of cervical cancer cells to stimulation with anti-CD95 agonist antibodies. We observed that treatment with low doses of IL-2 plus concentrations of CD95 that induce proliferation does not have a cumulative effect, that is, proliferation shows the same behaviour as independent stimuli in SiHa and HeLa cells (Figure 3C,D). However, when cervical cancer cells are stimulated with high doses of IL-2 and subsequently with anti-CD95 agonist antibodies at a concentration that induces proliferation, we observed that proliferation is recovered (Figure 3E,F), that is, the inhibition induced by IL-2 is lost.

### 3.4. Simultaneous Treatment with IL-2 and Anti-CD95 Does Not Induce an Increase in the Apoptotic Cells

**Figure 4:**
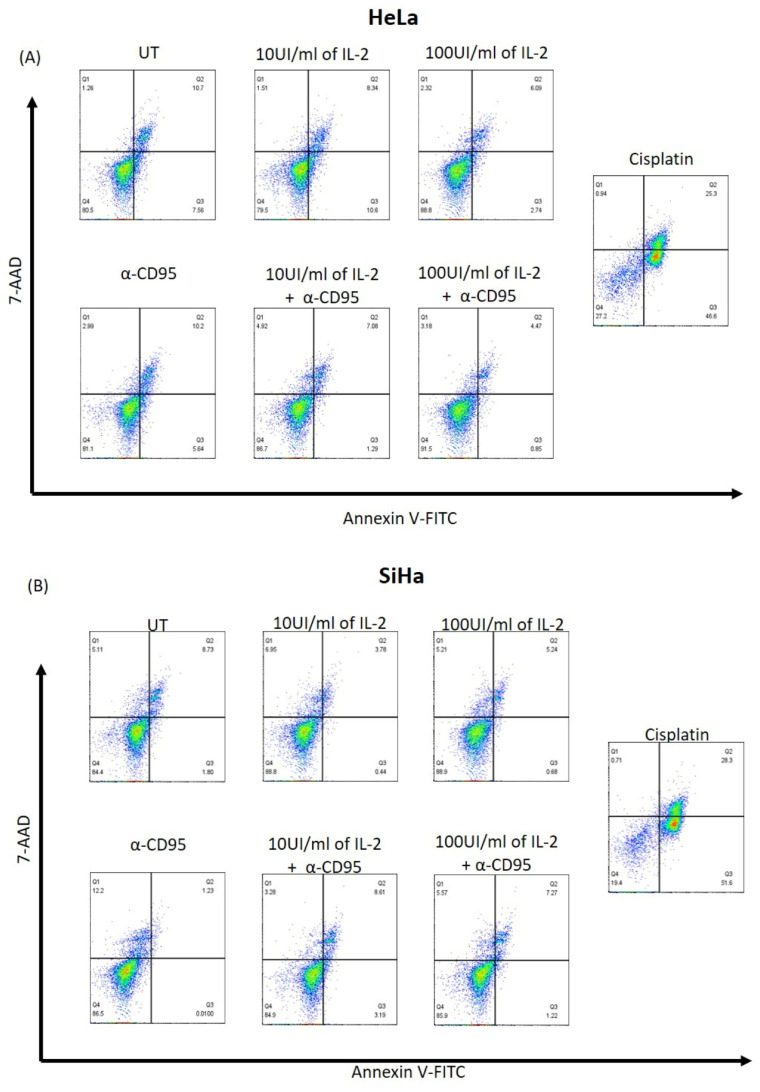
Determination of cell death by Annexin V/7AAD in HeLa and SiHa cells treated with IL-2, anti-CD95, and simultaneous treatments. Determination of apoptosis in (**A**) HeLa and (**B**) SiHa cells without treatment (UT), treated with 10 IU/mL of IL-2, 100 IU/mL of IL-2, anti-CD95, 10 IU/mL of IL-2 plus anti CD95, 100 IU/mL of IL-2 plus anti CD95, and, as a positive control, cells treated with cisplatin (IC50 2 μg/mL). Representative dot plots of three different experiments.

Because the activation of the CD95 pathway and sometimes the IL-2 pathway in cells of the immune system could induce an increase in apoptotic or autophagy cells, we decided to evaluate apoptotic cells with Annexin V and 7AAD, as well as the presence of LC3B and fluorescence foci that indicate autophagy. Independent or simultaneous treatment with IL-2 and low concentrations of the anti-CD95 agonist antibody in HeLa and SiHa cervical cancer cells did not impact the increase in apoptotic cells (Figure 4).

### 3.5. Simultaneous Treatment with IL-2 and Anti-CD95 Induces an Increase in Fluorescence for LC3B and in Fluorescent Foci

**Figure 5:**
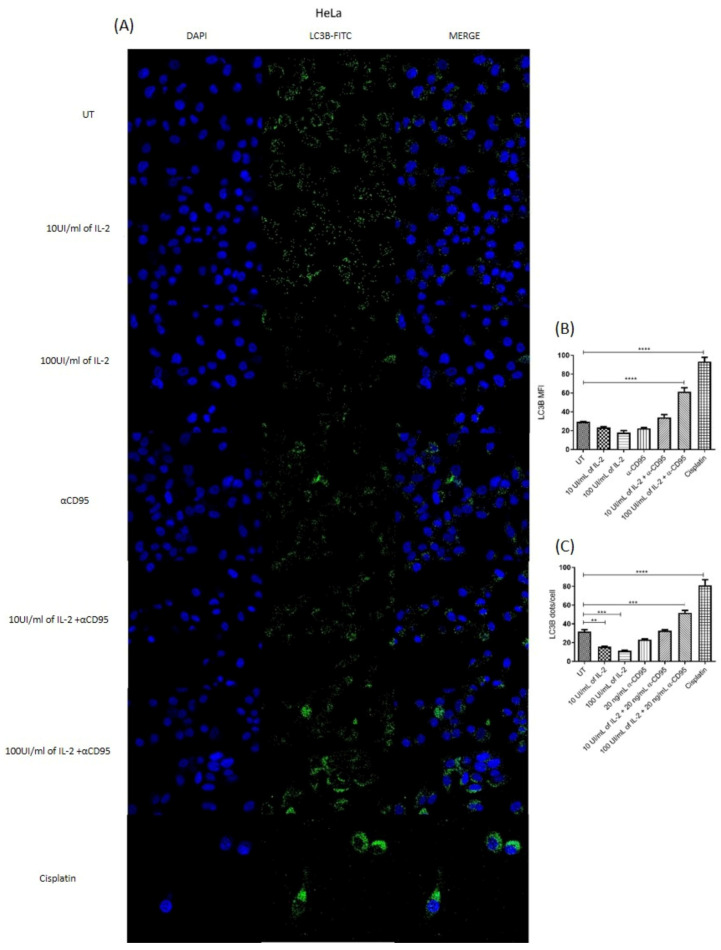
Determination of LC3B by confocal microscopy in HeLa cells. (**A**) Determination of LC3B in HeLa cells without treatment, treated with 10 IU/mL of IL-2, 100 IU/mL of IL-2, anti-CD95, 10 IU/mL of IL-2 plus anti-CD95, 100 IU/mL of IL-2 plus anti-CD95, and, as a positive control, cells treated with cisplatin (IC50 2 μg/mL). DAPI staining for nuclei, anti-LC3B FITC for autophagosomes, and a merging of fluorescence were performed. (**B**) Graph representing the average fluorescence intensity of LC3B, measured with the help of the Fiji software 2.9.0. (**C**) Graph of positive fluorescent foci for LC3B. ** *p* < 0.001, *** *p* < 0.0002, and **** *p* < 0.0001.

**Figure 6:**
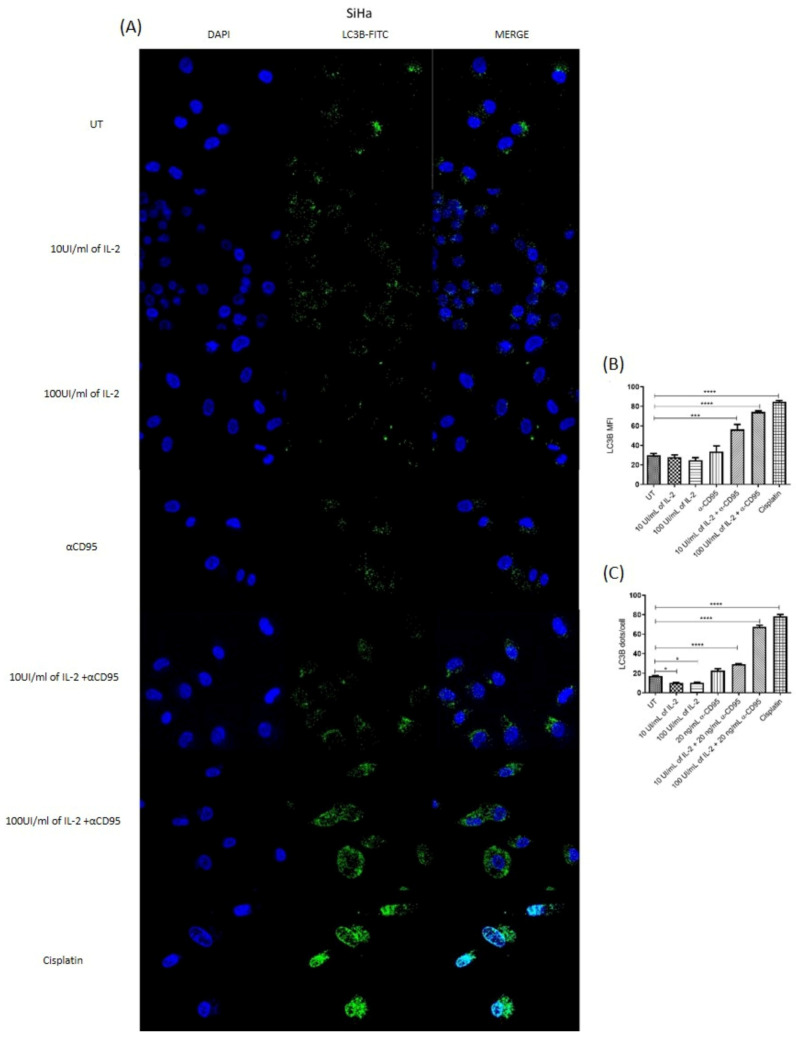
Determination of LC3B by confocal microscopy in SiHa cells. (**A**) Determination of LC3B in SiHa cells without treatment, treated with 10 IU/mL of IL-2, 100 IU/mL of IL-2, anti-CD95, 10 IU/mL of IL-2 plus anti-CD95, 100 IU/mL of IL-2 plus anti-CD95, and, as a positive control, cells treated with cisplatin (IC50 2 μg/mL). DAPI staining for nuclei, anti-LC3B FITC for autophagosomes, and a merging of fluorescence were performed. (**B**) Graph representing the average fluorescence intensity of LC3B, measured with the help of the Fiji software 2.9.0. (**C**) Graph of positive fluorescent foci for LC3B. * *p* < 0.01, *** *p* < 0.0002, and **** *p* < 0.0001.

Simultaneous treatment with high doses of IL-2 plus the anti-CD95 agonist antibody increases fluorescence for LC3B and in fluorescent foci in HeLa and SiHa cells (Figure 5 and Figure 6). However, these same treatments induce a recovery in proliferation, in which the activation of the apoptotic pathway is not observed. The fluorescence foci indicate a probable activation of a cytoprotective mechanism in cervical cancer cells.

## 4. Discussion

Tumour cells develop different mechanisms that favour their survival and proliferation. These cells affect different signalling pathways to avoid cell death. Interestingly, in this study, we focused on analysing two signalling pathways that can have pleiotropic effects: the CD95 and IL-2 pathways; an analysis of their response was carried out individually and in combination due to their behaviour. We had previously shown that HPV-positive cervical cancer cell lines express the death receptor CD95, while HPV-negative cell lines contain the receptor intracellularly [4]. In this study, we demonstrated that HPV18+ (HeLa) and HPV16+ (SiHa) cervical cancer cells not only express the death receptor CD95, but are also capable of expressing the CD95 ligand (Figure 1). Other reports have shown that HPV+ cervical tumour cells are highly resistant to the induction of apoptosis by the CD95 pathway because viral oncoproteins affect the signalling of this pathway [3,5]. However, it has recently been described in other types of cancers that the stimulation of the CD95 receptor can trigger the activation of a non-canonical pathway, which induces proliferation, metastasis, and different survival mechanisms [21,22,23]. Therefore, we investigated whether the activation of the CD95 pathway in cervical cancer cells could be involved in proliferation processes and whether they were sensitive to undergoing apoptosis. Our results indicate that the stimulation of HeLa and SiHa cells with different concentrations of anti-CD95 agonist antibodies (DX2 and CH11) has a differential effect: low concentrations induce cell proliferation, intermediate concentrations do not affect proliferation, and very high concentrations are needed to induce cell death (Figure 2). We also performed a morphological and cell size analysis, which showed that intermediate concentrations that do not affect cell proliferation did increase the size of the cells. We also observed that antibody concentrations that induce proliferation in tumour cells induce a slight increase in apoptosis in peripheral blood lymphocytes (manuscript in preparation). An interesting finding was that the induction of proliferation in HeLa and SiHa cells occurs at the same concentration of CD95. We expected to find a differential effect because a difference has been reported in the sensitivity between HPV18 and HPV16+ cells to the induction of apoptosis via CD95. In addition, it has been described that the viral oncoprotein E6 of HPV 16 can bind to FADD and thus promote the inhibition of apoptosis [3,5]. However, the data did not show differences between the HPV18+ and HPV16+ cell lines. The relevant contribution of this part of the study was to show that cervical tumour cells can use the CD95 receptor to induce their proliferation, and by knowing that they are also capable of expressing their ligand, one can speculate about probable autocrine stimulation. In cervical cancer and other types of solid tumours, the presence of CD95L has been related to resistance to cell death, evasion of the immune system, carcinogenesis, motility and invasiveness, and stemness; thus, it is essential in tumour maintenance and poor prognosis. Furthermore, it has been reported that the expression of CD95L in cervical tumours is related to the induction of apoptosis in lymphocytes infiltrating the tumour [6,7,9,24,25,26]. We show that cervical cancer cells can use the CD95 pathway to induce their proliferation; this effect could be due to the activation of signalling pathways such as NFκB and PI3K/MAPK, and likely the JAK/STAT pathway.

On the other hand, we previously demonstrated that HPV18+ cervical cancer cell lines (CALO, INBL, and HeLa) can respond differentially to treatment with low and high concentrations of IL-2, inducing or inhibiting their proliferation, respectively [12,13,27]. This work investigated whether this response was shown in the SiHa cell line (HPV16+). We observed that treatment with 10 IU/mL shows an increase in proliferation of up to 30% compared to the control and, in contrast to treatment with 100 IU/mL, induces a decrease in proliferation of up to approximately 30% (Figure 3A,B); it is important to mention that the decrease in proliferation is not due to an increase in apoptosis (Figure 4B). In addition, we have observed that the cervical cancer cell line C33A (HPV-negative) also responds to low doses of IL-2, increasing its proliferation, and to high doses, which decrease it (manuscript in preparation). These results indicate that IL-2 has a similar effect on cell proliferation regardless of HPV type. In HeLa cells, we previously reported that this decrease in proliferation was due to an arrest of the cell cycle in the G1 phase [13], which could contribute by increasing the sensitivity of the cells to the activation of apoptotic pathways. Considering that the CD95 and IL-2 pathways have differential effects on cervical tumour cells, we decided to evaluate whether simultaneous treatments affected the proliferation and induction of cell death. The results showed that the simultaneous treatment of proliferation-inducing concentrations of anti-CD95 and IL-2 in cervical tumour cells does not have a cumulative effect, which could suggest that both stimuli could be activating the same signalling pathways (Figure 3C,D). Interestingly, the results showed that when the cells are treated with high doses of IL-2 and their proliferation is decreased, the addition of anti-CD95 agonist antibodies induces a recovery in cell proliferation (Figure 3E,F), which was observed in both cell lines. Because high doses of IL-2 induce an arrest in the G1 phase of the cell cycle in HeLa cells, these cells could be more susceptible to activating apoptosis when treated with anti-CD95; however, none of the treatments induced an increase in apoptosis (Figure 4A,B).

The activation of the IL-2 and CD95 pathways has been associated with the activation of the autophagy pathway in lymphocytes, fibroblasts, and HeLa cells among other normal and tumour cell lines [15,17,28]; thus, we decided to analyse the presence of LC3B and fluorescence foci in the cells subjected to the different treatments. Interestingly, we observed that after the treatment with low and high doses of IL-2, there was a lower presence of fluorescence foci for LC3B, although the MFI was not modified (Figure 5 and Figure 6B,C). On the contrary, simultaneous treatments with 100 IU of IL-2 plus the anti-CD95 antibody increased the MFI for LC3B and fluorescent foci for LC3B; this effect was observed in both cell lines. However, this treatment is the one previously observed that induced the recovery of proliferation, and there was no increase in apoptosis, so we assumed that the increase in LC3B and the fluorescence foci were not related to the activation of the autophagy process, but instead to the activation of proliferation or perhaps a cytoprotective mechanism. It has previously been reported that the increase in LC3B in breast cancer, melanoma, hepatocellular carcinoma, and gastric cancer cells leads to an increase in cell proliferation [29,30,31,32]. Also, it is essential to mention that some studies in HeLa cells have described that the activation of autophagy induced by CD95 or TRAIL inhibits cell death [17,33]. Other studies on SiHa and HeLa cells show that autophagy reduces the cytotoxic effect of the drugs Ciclopiroxolamine and Pirarubicin [34,35], which suggests that the increase in LC3B and fluorescence foci in the joint treatment of high doses of IL-2 plus anti-CD95 agonist antibody could be associated with a cytoprotective effect. This study shows that HeLa and SiHa cervical tumour cells express the CD95 receptor and its ligand CD95L and can use this pathway to induce their proliferation. We did not find that the type of HPV in these cell lines affects this response. On the other hand, we show that the SiHa cell line (HPV16+) can also respond to treatment with low doses of IL-2 by increasing its proliferation and to high doses of IL-2 by decreasing its proliferation. Interestingly, we observed that the simultaneous treatment with concentrations of IL-2 and anti-CD95 that induce proliferation does not have a cumulative effect. However, the decrease in proliferation induced by high doses of IL-2 can be reversed by adding the anti-CD95 antibody; this restores the proliferation percentages and increases the mean fluorescence intensity for LC3B and the number of fluorescence foci compared to cells with only one treatment. This combination impacts the activation of apoptosis, which is why we propose that the simultaneous stimulus could activate a cytoprotective mechanism.

In this work, we observed how the cervical tumour cells HPV16 and 18 can use the IL-2 and CD95 pathways to induce their proliferation and survival. However, an interesting aspect to have in mind is that we do not know the effect of stimulating the CD95 and IL-2 pathways in in vivo models with cervical tumour cells. Another essential aspect to evaluate later would be the expression of CD95, CD95L, IL-2, and IL-2R in other types of cancer associated with the presence of HPV to determine whether these molecules are present and whether they are involved in cell proliferation. Furthermore, we demonstrated that stimulating the IL-2 and CD95 pathways with low cytokine and agonist antibody doses can induce tumour proliferation. Therefore, these data are very interesting and could suggest that blocking these pathways is more relevant to the biology of tumour cells. It would be worth evaluating its effect to determine whether it could have therapeutic importance.

## 5. Conclusions

This study shows that the death receptor CD95 and its cognate ligand are present in cervical cancer cells, and low doses of the agonist anti-CD95 antibodies induce cell proliferation. Treating cells with high doses of IL-2 inhibits cell proliferation, and simultaneous treatments with high doses of IL-2 and anti-CD95 agonist antibodies revert cell inhibition and increase LC3B foci in cervical cancer cells, but do not induce apoptosis. By simultaneously activating the CD95 and IL-2 signalling pathways, a cytoprotective mechanism leads to cervical cancer cell survival.

## Data Availability

The data presented in this study are available upon reasonable request from the corresponding author.
